# Loss of Motility as a Non-Lethal Mechanism for Intercolony Inhibition (“Sibling Rivalry”) in *Marinobacter*

**DOI:** 10.3390/microorganisms9010103

**Published:** 2021-01-05

**Authors:** Ricardo Cruz-López, Piotr Kolesinski, Frederik De Boever, David H. Green, Mary W. Carrano, Carl J. Carrano

**Affiliations:** 1 Department of Chemistry and Biochemistry, San Diego State University, San Diego, CA 92182-1030, USA; ricardo.crlp@gmail.com (R.C.-L.); pkolesinski82@gmail.com (P.K.); mwcarrano@yahoo.com (M.W.C.); 2 SAMS, Scottish Marine Institute, Oban, Argyll PA37 1QA, UK; frederik.deboever@sams.ac.uk (F.D.B.); david.green@sams.ac.uk (D.H.G.)

**Keywords:** sibling rivalry, *Marinobacter*, bacteriocins, quorum sensing, motility

## Abstract

Bacteria from the genus *Marinobacter* are ubiquitous throughout the worlds’ oceans as “opportunitrophs” capable of surviving a wide range of conditions, including colonization of surfaces of marine snow and algae. To prevent too many bacteria from occupying this ecological niche simultaneously, some sort of population dependent control must be operative. Here, we show that while *Marinobacter* do not produce or utilize an acylhomoserine lactone (AHL)-based quorum sensing system, “sibling” colonies of many species of *Marinobacter* exhibit a form of non-lethal chemical communication that prevents colonies from overrunning each other’s niche space. Evidence suggests that this inhibition is the result of a loss in motility for cells at the colony interfaces. Although not the signal itself, we have identified a protein, glycerophosphoryl diester phosphodiesterase, that is enriched in the inhibition zone between the spreading colonies that may be part of the overall response.

## 1. Introduction

*Marinobacter* belong to the class of γ-proteobacteria and these motile, halophilic, or halotolerant bacteria all share the ability to use petroleum hydrocarbons as their sole energy and carbon sources [1]. Numerous strains of *Marinobacter* have been described from ecological niches as diverse as high-latitude seawater and Antarctic sea ice [2,3,4], hypersaline mats [5,6], oil-producing wells [7], oil-contaminated marine sediments [8], and even marine sponges [9]. In addition, this genus is often found in association with phytoplankton [10,11,12,13,14,15]. Because of its wide ecological distribution, the *Marinobacter* genus is ubiquitous in the ocean and its species can be described as “opportunitrophs” able to survive under a wide-range of conditions [16].

While *Marinobacter* spp. account numerically for only a small proportion of the total bacteria present in the “phycosphere” of algae-associated environments, either in culture or in the field [17], they are almost always present in the core microbiome of phytoplankton in general and dinoflagellates in particular [10,18,19,20,21]. Thus, they appear to be a “keystone” taxon that may modulate the physiology of the host via iron acquisition through the secretion of iron siderophores [22], inducing cell aggregation [23,24], or excystment through the release of growth factors [25,26].

Although many potentially algal-associated bacteria occur in seawater, it is perhaps surprising that in general only a few individuals, on average 1.8–2.3 [18,27], actually colonize the surface of a phytoplankton host at the same time, suggesting a possible intraspecies competition for this niche. In a more general sense, all bacterial communities compete for resources and niche space. This would include colonization of algal surfaces as part of various types of bacterial–algal interactions. Thus, there must be various ways that bacteria and/or algae can control the colonizing population of an algal surface, most of which likely invoke some sort of cell-to-cell communication. Indeed, previous studies have demonstrated that cell-to-cell communication across populations is concentrated within marine microhabitats such as biofilms, marine snow, and eukaryotic co-associations [28].

Currently the most well-studied and best-understood mechanism of bacterial communication is quorum sensing based on N-acyl homoserine lactone (AHL) signaling, which is utilized by Gram-negative bacteria within the alpha-, beta-, and γ-proteobacteria classes [29]. Cells within individual populations synthesize diffusible AHL molecules, the concentration of which increases proportionally with increasing population density until a critical threshold concentration is reached, at which time the AHL binds to receptor proteins responsible for the transcription and subsequent expression of target genes encoding for a particular phenotype [29,30]. Quorum sensing dependent phenotypes include antibiotic production, biofilm formation, production of virulence factors, and swarming motility [28,30,31]. These behaviors are particularly important for successful colonization of higher organisms by opportunistic pathogens such as *Pseudomonas aeruginosa* and *Vibrio harveyi* [32]. The *Marinobacter* group is phylogenetically similar to *Pseudomonas* [7,33], however, there is only limited evidence that *Marinobacter* are capable of producing AHLs [34,35]. Thus, it is presently unknown whether AHL synthesis or utilization is a general characteristic of the genus.

A parallel approach to controlling population density in bacterial communities is through the excretion of cell-to-cell signals that function as “no trespassing” or “no-go zone” signs such as bacteriocins [36]. Bacteriocins are biologically active small peptides or protein complexes that display an allelopathic or bactericidal mode of action towards the same or closely related species [37] and have been found in all major lineages of bacteria [38]. They may also serve as anticompetitors, enabling the invasion of a strain or a species into an established microbial population or community, or play a defensive role and act to prohibit the invasion of other strains or species into an occupied niche or limit the advance of neighboring cells [39,40]. The abundance of bacteriocin-producing genotypes is assumed to be a consequence of competition for limited resources [38], and it is expected to occur more frequently between closely-related genotypes, which compete for the same resource, and less frequently between unrelated genotypes [41] or between intermediate genotypes [42]. Bacteriocin producers frequently invade and outcompete sensitive competitors; however, it remains unclear why bacterial genotypes inhibit some competitors and not others, and how the bacteriocin-mediated interactions influence the distribution of genotypes in free-living bacterial communities. Bacteriocin activity has been documented in at least some marine bacterial isolates [43], however, the evidence for more general representation among marine bacteria is lacking. Thus, here, using strains of the *Marinobacter* genus isolated from marine phytoplankton, we screen for both AHL production and for bacteriocin-like inhibitory substances (BLIS) to explore their possible roles in niche competition.

## 2. Materials and Methods

### 2.1. Strains, Growth Media, and Conditions

Bioreporter strains were obtained from Prof. Robert McLean, Texas State University. *Chromobacterium violaceum* is a mini-Tn5 mutant strain of *C. violaceum* that produces the purple pigment violacein in response to QS AHLs (C4–C8), while *Chromobacterium violaceum* strain 31,532 is a C6-AHL overproducer and serves as a positive control for AHL detection. Strain 12,472 is the wild type and is used to detect potential quorum signal inhibitors via a loss of the characteristic purple pigmentation. A136 is a strain of *Agrobacterium tumefaciens* that contains a lacZ reporter and responds to the presence of medium *Agrobacterium tumefaciens* A136 (pCF218). (pCF372) is a bioassay strain for a wider range of medium-to-long chain (C6-C10) AHLs [44]. KYC6 is a 3-oxo-C8 AHL overproducer and is used as a positive control for the A136 AHL bioassay. *C. violaceum* and *A. tumefaciens* bioreporters were routinely grown at 30 °C in Luria broth medium (LB; 10 g/L tryptone, 5.95 g/L yeast extract, 10 g/L NaCl, pH 7.0). As required, LB media was supplemented with bioreporter-specific antibiotics at the reported working concentrations.

*Marinobacter algicola* DG893 (hereafter DG893) was cultivated on either marine broth (hereafter MB; 5 g/L peptone, 1 g/L yeast extract, 75% *v/v* natural sea water, pH 7.5) or artificial sea water (hereafter ASW; 15 g/L NaCl, 0.75 g/L KCl, 1.0 g/L NH_4_Cl, 12.4 g/L MgSO_4_·7H_2_O, 3.0 g/L CaCl_2_·2H_2_O, 0.1 g/L β-glycerophosphoric acid, 5 g/L sodium succinate, pH 7.5) at 25 °C. Liquid cultures were grown at 25 °C with constant shaking at 150 rpm for 3 days in sterilized 25 mL test tubes. Cultures grown in liquid broth for screening and extraction purposes and were agitated at 200 rpm in a rotating incubator set to the appropriate temperature to ensure sufficient oxygenation of the sample. Working agar plates were made with the same media containing 1.5% agar. For motility-related experiments, media were solidified with 0.25% agar. Afterwards, 5 µL aliquots of stationary phase culture were spotted onto plate surfaces and allowed to grow diffusively until the desired area coverage was achieved. For viability tests, 5 mL of melted medium containing 0.25% agar was inoculated with stationary phase culture in a 1:100 ratio and overlaid onto precast agar plates solidified with 1.5% agar.

### 2.2. AHL Bioassays

While initial attempts to grow the bioreporter strains on marine broth media were unsuccessful, *Marinobacter* were found to grow reasonably well on LB plates, hence the latter were used for most assays. However, since it was possible that *Marinobacter* grown on the non-ideal LB media might give different quorum sensing (QS) signals as compared to cells grown on the more natural marine broth medium, we also developed a split plate assay since the classical overlay method [44] did not give reproducible results in our hands. Ultimately, no differences in results were apparent between the two methods. Bioassays using CV026 utilized the classical T-streak plate method with formation of the purple pigment at the intersection of the streaks, indicative of production of C4–C6 AHLs by the test strain. Bioassays utilizing *A. tumefaciens* A136 were conducted in the same was as those for CV026 except that the plates used were first spread with 50 µL of filter-sterilized 10 mg/mL stock solution of X-gal in dimethylformamide prior to inoculation, where a positive response was the formation of a blue-green color around the reporter strain.

### 2.3. AHL Extraction from Marinobacter Supernatants

Methods of extraction were based on those described by Twigg et al. [45]. Individual *Marinobacter* strains (29 in total) were inoculated in 10 mL of marine broth and incubated for 72 h. Cells were pelleted by centrifugation at 8000 rpm for 15 min, with subsequent decanting of the supernatant into acid-washed glass culture tubes. AHLs were extracted from acidified supernatant by adding 5–25 mL of dichloromethane or ethyl acetate and placing culture tubes in an orbital shaker for 1–2 h to allow for phase mixing. The organic phase removed by either rotary evaporation or dried using nitrogen gas, was reconstituted in 0.5 mL acetonitrile and transferred to glass vials. Extracts were stored at −20 °C until assayed. Negative controls were produced by performing the extraction procedure on sterile MB and LB media.

### 2.4. In Silico Search for Putative AHL Biosynthetic Genes

The Integrated Microbial Genome (IMG) system was used for the analysis of draft and complete microbial genomes [46]. Previous reviews of LuxR regulatory apparatus and N-acyl homoserine lactone formation [30,32] facilitated the search for genes of potential interest in *Marinobacter algicola* DG893 (described here as a model genome). For the determination of putative functions, IMG-generated gene annotations were examined. Clusters of orthologous groups of proteins (COGs) and functional protein categories (Pfam) were the principal annotations focused upon throughout this study. Where required, BLASTp was used to compare proteins [47].

### 2.5. Bacteriocin-Like Inhibitory Substance (BLIS) Assay

The agar plates were inoculated by placing 10-μL droplets of a liquid culture on the surface. For intercolony competition experiments, 2 droplets were inoculated 1–3 cm equidistant from the center, along a line through the plate’s center. The plates were maintained at 25 °C in the dark. Images were obtained with a 14.7-megapixel Canon G10 camera with a 6.1–30.5-mm lens. The camera was placed above the plates at a distance of 10 cm. Each petri dish was mounted above a white transilluminator.

### 2.6. Metabolic and Cell Viability Assays

For evaluation of succinate dehydrogenase activity of motile cells, agar plates were covered with 10 mL of 0.01% triphenyltetrazolium chloride (TTC) and incubated for ca. 30 min at 25 °C until a red color resulting from formazan precipitation was developed. Peroxidase activity was assessed in a similar way but a 0.05% diaminobenzidine (DAB)/0.015% hydrogen peroxide solution was used as an overlay instead. Full brownish color development was reached after 1–24 h. Cell viability was tested on low-concentration agar plates that were supplemented with 0.5 µM propidium iodide (PI) and cultivated at 25 °C in the dark. Accumulation of dye by dead or dying cells led to increased red fluorescence observed under UV transillumination as PI can only bind to the DNA of dead or membrane-compromised bacteria as it is impermeable to living cells. For additional cell viability determination, growing colonies of bacteria were harvested with a toothpick in the colony interior and at the edges of the motility zone facing either sterile agar or a competitive colony. For each zone of interest, *n* = 6 probing spots were used. Cells on the tip of the toothpick were used for inoculation of 200 µl fresh MB medium and allowed to grow with gentle agitation for 24 h at 25 °C. Afterwards, bacteria turbidity was measured as OD600. Ability of cells at the interface to regain motility was assayed by harvesting cells at the edge of the inhibition zone and far away from it (control) using a sterile toothpick and respotting them onto fresh 0.25% ASW agar plates.

### 2.7. Isolation of Active Compounds Secreted by DG893

Solid medium from the motility-inhibition zones as well as from the area adjacent to single motile colonies was excised, fragmented by repetitive pipetting, and frozen for 2 h at −80 °C. Afterwards, samples were thawed at room temperature and debris containing dehydrated agar chunks as well as residual bacterial cells was removed by centrifugation at 15,000× *g* for 15 min at 4 °C. Supernatant was collected and either subjected to ammonium sulfate precipitation to 80% of saturation or extraction with ethyl acetate. Ammonium sulfate precipitate was harvested by centrifugation at 15,000× *g* for 15 min at 4 °C and re-dissolved in PBS.

### 2.8. Protein Identification

Ammonium sulfate precipitate containing proteins secreted by motile bacteria was separated by 12% Tris-Glycine SDS-PAGE and stained with Coomassie Brilliant Blue R-250. A single dominant protein band was observed with an apparent molecular weight of approximately 40 kDa. This band was excised by razor blade, subjected to in-gel trypsin digestion, and identified by mass spectrometry. Mass spectrometry-based peptide identification was performed by the Biomolecular and Proteomics Mass Spectrometry Facility at University of California San Diego (San Diego, CA, USA).

### 2.9. Microscopic Sample Preparation and Imaging

Fluorescence microscopy was performed on a DG893 film generated from a starting culture at exponential phase (0.5 OD600). Two 1 μL droplets were spotted 0.5 cm away from each other on a 7.5 × 2.5 cm glass slide covered with a thin layer of 500 μL of semisolid MB (0.3% agar). Incubation was carried out at 25 °C in a petri dish (100 mm × 15 mm, Celltreat) containing two pieces of filter paper #1 (60 mm × 20 mm, Whatman) soaked with 100 μL of deionized sterile water and sealed with parafilm to avoid agar desiccation. After three days of incubation, an 11 × 22 mm area from the semisolid agar was removed from both edges and replaced by hybridization cover slips (11 × 22 mm, Grace Bio-Labs), stained with 1 μL SYBR Green I (10,000 × stock, Invitrogen) and 0.5 μL propidium iodide (20 mM) in 28 μL of antifading solution [48]. This was then covered with a micro cover glass (24 × 50 mm, VWR). Visualization was conducted on a BZ-X810 Keyence all-in-one fluorescence microscope, using dual band excitation and emission FITC-TRITC filters, at 60× magnification.

## 3. Results

### 3.1. Quorum Sensing (QS)

To determine if AHLs were being produced by the various species of *Marinobacter,* we used two different bioreporter strains, CV026 and A136, which both failed to show evidence for the production of either long or short chain homoserine lactones. In addition, all attempts to isolate and characterize any AHLs from cultures of 40+ species of *Marinobacter* using standard extraction and LC-MS methods failed to yield any evidence for their existence. Spiking *Marinobacter* media with small quantities of known AHLs followed by extraction clearly revealed the presence of the AHL by LC-MS, indicating that the failure to identify any AHL in those *Marinobacter* species tested did not arise from an inadequacy in the extraction and characterization methodology but rather from the actual lack of AHL production. To further support the absence of an AHL based QS system, we searched for putative AHL biosynthetic genes in several of the available *Marinobacter* genomes. The typical Gram-negative QS system contains LuxR proteins, which upon binding to associated AHL autoinducer molecules induce transcription of target genes [30]. LuxR proteins function in response to LuxI proteins, which synthesize AHLs from S-adenosylmethionine substrate [30,32]. However, consistent with our bioreporter and AHL isolation results, we could find no homologous LuxI genes within the *Marinobacter* genomes, reinforcing the idea that an AHL-based QS system was not present.

### 3.2. Intraspecies Interactions

**Figure 1:**
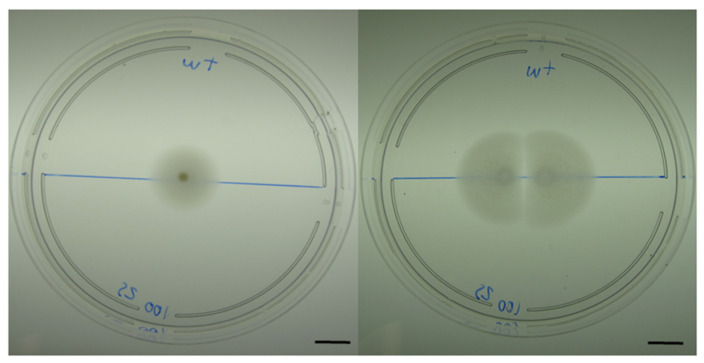
*M. algicola* DG893 growth on low percentage marine agar (0.25%) plates. The left pane shows growth of a single colony expanding by swarming while the right pane shows the growth of two sibling colonies placed a few centimeters apart, showing a clear zone of inhibition where the two colonies approach one another. Scale bar: 1 cm.

To search for intraspecies bacteriocin/toxin effects we spotted 5 µL of liquid cultures of various *Marinobacter* species (Table S1) on low percentage agar plates that allow for colony expansion via swimming or swarming. When only a single aliquot of motile *M. algicola* were spotted onto 0.25% agar plates (hereafter agar plates), the colony expanded evenly in a radial manner. However, when sibling colonies were spotted near each other (within a few cm), they also grew radially until the colony edges came close to one another (>1 cm apart) whereupon expansion ceased with formation of sharp inhibition zones at these interfacial areas (Figure 1). We initially hypothesized that colonies nearing each other were producing toxins such as the described “sibling lethal factor” [49,50], which would result in the killing of cells at the interface.

**Figure 2:**
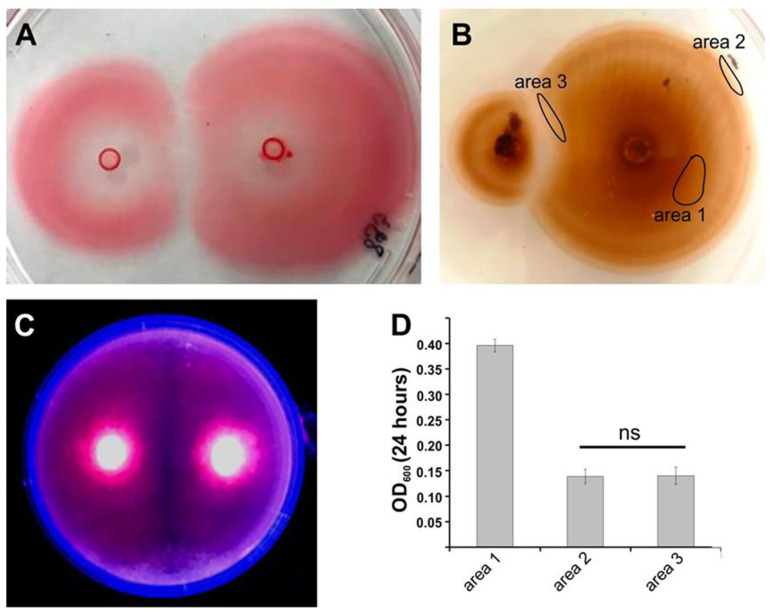
Metabolic activity and viability of motile *M. algicola* DG893 cells. To assess whether the motile cells were metabolically active. Agar plates were overlaid with either triphenyltetrazolium chloride (**A**), or diaminobenzidine and H_2_O_2_ (**B**), to visualize succinate dehydrogenase or peroxidase enzymatic activity, respectively. Non-stained regions are considered as metabolically inactive. Propidium iodide (PI) is known to incorporate into dead or permeable cells, thus the lack of staining shows that the cells near the inhibition zone are still viable (**C**). To verify viability of cells from different zones (marked on section B), their growth after 24 h was measured by OD600. For each area, mean ± SD for *n* ≥ 5 probing spots are presented (**D**); ns, not significant.

To assess whether the cells in the inhibition zone were dead, or viable and still metabolically active, we utilized various enzymatic tests, a vital dye incorporation assay, as well as grow-out experiments (Figure 2). 

**Figure 3:**
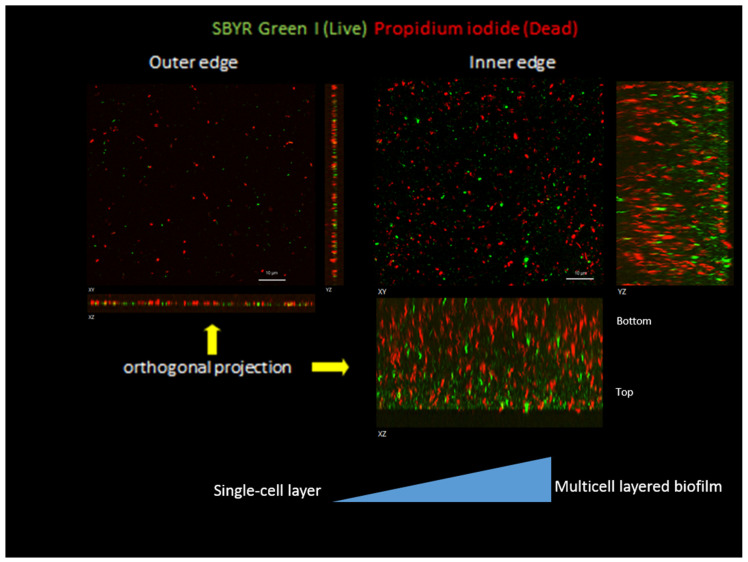
Images of *M. algicola* DG893 intercolony film on a thin layer of 0.3% agar, observed under fluorescence microscopy using SYBR Green I (green) and propidium iodide (red) visualized by FITC and TRITC filters to detect cell viability. Scale bar: 10 μm.

Positive succinate dehydrogenase or peroxidase activities were visualized by TTC or DAB staining, respectively. The red color of cells adjacent to the inhibition zones resulting from formazan precipitation indicated an unperturbed Krebs cycle, while lack of staining of corresponding areas with DAB suggest a lack of peroxidase activity in cells adjacent to inhibition zones. Propidium iodide (PI) staining was observable only in the cells located in “old” areas close to the center of the colony, indicating a loss of cell viability in this region. Cells at the edges of the expanding colony remained unstained and were assumed to be viable. Cell viability was independent of the location around the colony periphery, and included those cells found at the edge of the inhibition zone. Thus, when bacteria picked from the edges of the inhibition zones or from areas far from it were cultured in marine broth, they reached similar optical density regardless of location. Finally, fluorescence microscopy of vital dye treated cells (SYBR Green and PI) positioned at the inhibition zone and at the edges of non-interacting zones confirmed that they were still viable with a constant ratio of live vs. dead cells at the interface between colonies and on the non-interacting edges of the colony, and dividing cells were still evident at the interface (Figure S1). Z-stack plots across a transect also revealed that at the edge of the colony opposite the interaction area, the colony spreading zone was only a few cells thick, while at the inhibition zone it appeared to be a biofilm hundreds of cells thick (Figure 3). This behavior is consistent with the hypothesis that the cells at the inhibition zone continue to divide but have lost motility. That the apparent motility inhibition was temporary was indicated by the fact that when cells from the inhibition zone were respotted onto fresh 0.25% ASW agar plates, they expanded radially at a rate similar to those of the controls, indicating motility of the cells from the interface was no longer impaired. Thus, rather than expanding out radially to new territory, they simply grow on top of each other in a kind of biofilm. Taken together the data suggests that rival sibling colonies initiate an inhibition of motility preventing encroachment into each other’s space rather than the mutual killing-off of potential competitors

### 3.3. Identification of Potential Bioactive Material

**Figure 4:**
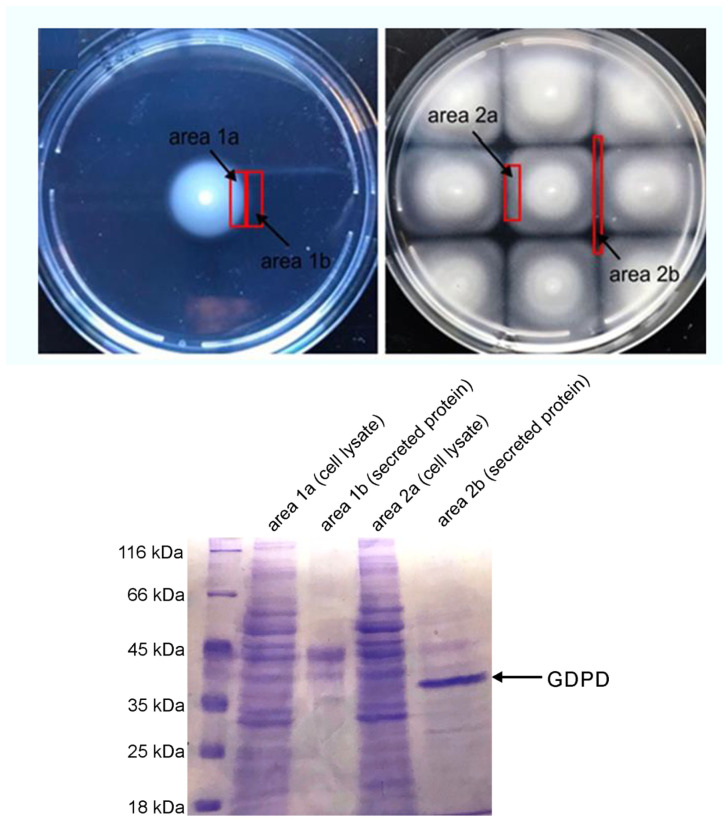
Secretion of proteins by colonies of *M. algicola* DG893. Upper left: Zones sampled (1a and 1b) for non-interacting colonies. Upper right: Zones sampled (2a and 2b) for interacting colonies. Lower: 12% SDS-PAGE separation of proteins from cell lysates or secreted proteins from indicated areas produced by *M. algicola*. Migration position of periplasmic glycerophosphoryl diester phosphodiesterase (RefSeq WP_007154889.1; GenBank EDM46611.1) is indicated with an arrow.

To assess the molecular basis of such behavior we attempted to isolate potential small molecules and/or secreted proteins from above the aforementioned inhibition zones. Despite extensive attempts that continue to this day, we have been thus far unable to isolate and characterize a potential small molecule signal associated with the motility loss. However, from the inhibition zone extract we observed a single major protein band upon ammonium sulfate precipitation and analysis via SDS-PAGE gel with an apparent molecular weight of approximately 40 kDa (Figure 4). No such protein could be visualized from extracts near the colony edge away from the inhibition zone. After in gel trypsin digestion and mass spectral analysis, the inhibition zone protein was identified as glycerophosphoryl diester phosphodiesterase (hereafter GDPD) with accession number WP_007154889.1. Analysis of the deduced amino acid sequence showed that the *M. algicola* DG893 GDPD contained 439 amino acids with an expected molecular weight of 49,314 Da, including a putative 21 amino acid N-terminal signal peptide [51,52] containing an alanine and a double leucine motif indicative of a secretory transport (Figure S2).

### 3.4. GDPD Network Analysis

To determine what, if any, relationship there was between the isolated GDPD protein and the apparent loss of motility of cells at the intercolony interface, we used the STRING Database v11.0 [53] to establish possible functional interactions among related proteins. STRING is a database of known and predicted protein–protein interactions. The interactions include direct (physical) and indirect (functional) associations; they stem from computational prediction, from knowledge transfer between organisms, and from interactions aggregated from other (primary) databases. In this analysis, the STRING Database predicted ten potentially interacting proteins (Table 1).

As illustrated in Figure S3, GDPD could be interacting with a FAD dependent oxidoreductase, a GDPD with six transmembrane domains, a glycerol-3-phosphate dehydrogenase (GPDH), two glycerol kinases, a phosphodiesterase/alkaline phosphatase D, a flagellar secretion chaperone FliS, an extracellular nuclease, a phosphatase, and a chemotaxis protein histidine kinase. Importantly, the STRING Database revealed an interaction between periplasmic GDPD and a chemotaxis protein histidine kinase that enables bacteria to sense, respond, and adapt to a wide range of environments, stressors, and growth conditions (http://www.ebi.ac.uk/interpro/entry/IPR014310), as well as potentially controlling a flagellar secretion chaperone protein present in this network. On the other hand, the STRING Database established a potential interaction of GDPD with two glycerol kinases involved in glycerol degradation (https://www.uniprot.org/uniprot/A6F2B1). Another interesting potential interaction was with an extracellular nuclease that is involved with diverse processes of a typical biofilm architecture in *Vibrio cholerae* [54].

Based on motility assays, however, it is clear that GDPD, in and of itself, is not the trigger for motility loss, as cells treated with partially purified protein continue to be motile. Taken together with some preliminary here, these data suggest that GDPD might exert an effect on interacting sibling colonies by triggering changes in triglyceride or glycerolphospholipid metabolism to have a role in biofilm formation. Nevertheless, detailed evaluation of any potential GDPD-related mechanism will have to await isolation and characterization of larger amounts of functional protein.

## 4. Discussion

Our initial goal was to search for, and identify, potential AHL QS molecules from bacteria of the genus *Marinobacter* that could be involved in cell–cell signaling of potential importance to population-dependent colonization of algal or other surfaces such as marine snow. As γ-proteobacteria are common producers of AHLs we were surprised to find only a very few examples in the literature [35] of a *Marinobacter* isolate as a possible AHL producer and no examples from our own screening of 40+ laboratory cultures of *Marinobacter* species. Searching the available *Marinobacter* genomes also failed to provide any evidence for the presence of AHL receptors or biosynthetic machinery. Thus, we conclude that most, if not all, members of this genus do not produce, nor utilize, an AHL-based QS system.

In the absence of a clearly defined QS system, we looked for alternate means by which bacterial populations in a particular ecological niche could be controlled and this led us to search for the production of bacteriocin-like substances by *Marinobacter*. Thus, when two sibling colonies of *M. algicola* DG893 were plated near each other on a soft nutrient agar plate, the colonies expanded isotropically until a well-defined distance was reached when colony expansion halted at the interface between the two colonies, forming a demarcation line (DL) or zone of mutual inhibition. Using a range of *Marinobacter* isolates, including our model *M. algicola* DG893, we repeatedly observed intercolony inhibition between sibling colonies (Table S1). It should be noted that intercolony inhibition was not restricted to “siblings” but was also observable between different species of *Marinobacter*. However, it is not clear that the mechanism is the same and this will be the subject of further investigation.

Superficially similar phenomenon between genotypically identical colonies growing on solid media where two types of behavior (merging vs. formation of DL) have been described by many authors [55,56,57,58,59] and a number of mathematical models that mirror this behavior have been presented. In most of these examples, colonies are found to merge when the agar concentration was low and to form a DL when the agar concentration was high, with nutrient depletion being the primary driver. Thus, it was proposed that rapidly expanding colonies (i.e., those growing on low percentage agar and moving rapidly via swimming or swarming) never have a chance to experience nutrient depletion before merging, while those expanding more slowly on high percentage agar have time to deplete the nutrients between colonies and thus stop growing at the interface, forming a DL. However, only in a few cases was a biochemical mechanism to account for this latter effect have been elucidated, such as *P. dendritiformis*, which produces and releases a protein termed “sibling lethal factor” (Slf), resulting in the killing of cells at the interface between colonies [49,50], or *M. xanthus,* which uses the type VI secretion system (T6SS), based on cell susceptibility to an immunity protein TsxEI [60]. Very recently, a non-lethal sibling rivalry has been described in *P. mirabilis*, a robustly swarming pathogenic bacterium, where an Ids self-recognition system selectively induces non-self cells into a growth-arrested lifestyle incompatible with cooperative swarming [61].

In order to determine if the inhibition we saw here was the result of a mutually lethal mechanism such as has been described for a “sibling lethal factor”, we employed a variety of tests to determine the viability of cells in the inhibition zone. However, unlike the situation with “sibling lethal factor”, all the data points to the cells at the inhibition zone remaining viable and metabolically active. Simple depletion of nutrients at the interface seems unlikely to be the cause of this effect since qualitatively and quantitatively similar inhibition effects are observed when sibling colonies of DG893 were grown on minimal media (ASW succinate) or rich media (MB) and the effect was reversed from that of previous studies in that a DL is formed by rapidly expanding colonies on low percentage agar. Indeed, we did not generally observe merging between sibling colonies of DG893 no matter what media was used. As the rival expanding colonies never come in contact with each other and nutrient depletion seems an inadequate explanation, some form of non-lethal chemical communication between the rival colonies seems likely. In an effort to search for chemical cues, extracts of the inhibition zone between colonies revealed the presence of a single protein that was absent in the agar from expanding non-interacting areas of the colonies.

In gel trypsin, digestion followed by mass spectral analysis identified the single dominant protein as a glycerophosphoryl diester phosphodiesterase (GDPD) similar to *Escherichia coli* periplasmic phosphodiesterase (GlpQ, NCBI entry WP_007154889.1). Here, GDPD is clearly being secreted into the external media, however, its role in the process remains unclear. Secretion of related phosphodiesterases into the media have also been reported during phosphate starvation in *B. subtilis* [62]. It is also notable that other phosphodiesterases such as DipA from *Pseudomonas aeruginosa* have previously been implicated in flagellum-mediated swimming and swarming [63]. Secreted GDPD could thus be considered as a type of bacteriocin in that it is a biologically active protein complex that may display an allelopathic mode of action towards the same or closely related species. However, GDPD has also been connected with bacteriocin resistance in *Enterococcus* [64].

As a relatively large protein, GDPD is likely to be the result of a signal rather than being a signal itself and, therefore, is probably only one part of a coordinated response to threat, population density, or nutrient depletion. Indeed, it is clear that the protein itself does not inhibit motility. If GDPD is not the signal, what then is the chemical signal that turns off motility? We hypothesize that environmental cues detected by bacteria are transduced into intracellular second messengers, such as cyclic di-GMP, (p)pGpp, or c-AMP, that in turn initiate the appropriate cellular responses, including a reduction or loss of motility [65]. Indeed, previous work [66] has established that that cyclic di-GMP levels are correlated with the transition between motile and biofilm phases, therefore, further work will seek to determine cyclic di-GMP levels to help elucidate the mechanism underpinning this switch in behavior.

Finally, “sibling rivalry” seems to be a widespread phenomenon among the *Marinobacter* genus, potentially enabling cells to discriminate between siblings. To our knowledge, this is the first documentation of “sibling rivalry” in marine bacteria, the mechanism of which differs from that previously reported. In *M. algicola* DG893, the inhibition is clearly non-lethal and appears to operate via reduction in motility. Such a non-lethal mechanism might regulate colonization of niches such as algal surfaces by a physiologic response that simply prevents two populations from physically occupying the same niche, and thus prevents wasteful resource competition between sibling cells.

## 5. Conclusions

The following points summarize the main conclusions of this work:

(1) Most if not all of the bacteria in the genus *Marinobacter* neither produce nor utilize either of the two main QS systems (i.e., those based on acylhomoserine lactones or alternatively AI-2) commonly found in Gram-negative bacteria.
(2) Many bacteria of the *Marinobacter* genus display clear and unambiguous sibling intercolony inhibition.
(3) The intercolony inhibition is not lethal.
(4) The inhibition between colonies is likely due to a loss in motility of the cells at the intercolony interface, and such motility is not permanent and is restored when cells are placed on fresh media.
(5) A secreted protein related to GDPD may be part of this process.

## Figures and Tables

**Table 1:** *M. algicola* glycerophosphoryl diester phosphodiesterase (GDPD) potential protein interactions based on the STRING database.

Node	Annotation	Acc. Number	Score
EDM46611.1	Glycerophosphoryl diester phosphodiesterase	MDG893_19429	Query
EDM49251.1	FAD dependent oxidoreductase	MDG893_07635	0.890
EDM47432.1	Glycerophosphoryl diester phosphodiesterase	MDG893_00180	0.877
gpsA	Glycerol-3-phosphate dehydrogenase (GPDH)	MDG893_06880	0.807
EDM47089.1	Glycerol kinase	MDG893_11889	0.756
EDM45841.1	Glycerol kinase	MDG893_05159	0.756
EDM46512.1	Phosphodiesterase/alkaline phosphatase D	MDG893_20089	0.754
EDM47357.1	Flagellar secretion chaperone protein FliS	MDG893_01080	0.724
EDM46845.1	Extracellular nuclease	MDG893_17732	0.721
EDM47042.1	Predicted phosphatase	MDG893_02110	0.712
EDM47259.1	Chemotaxis protein histidine kinase	MDG893_18974	0.681

## Supplementary Materials

The following are available online at https://www.mdpi.com/2076-2607/9/1/103/s1, Table S1: Sibling colony inhibition in different strains of the *Marinobacter* genus isolated from marine environments. Figure S1: *M. algicola* DG893 biofilm growing on 0.3% MB agar, in a horizontal transect, Figure S2: Sequence analysis of the GDPD signal peptide by SignalP 5.0 server, Figure S3: *M. algicola* DG893 GDPD protein–protein interaction network based on the STRING Database.
