# Supplementary material for: Loss of Motility as a Non-Lethal Mechanism for Intercolony Inhibition (“Sibling Rivalry”) in Marinobacter
Source: Microorganisms. 2021 Jan 5;9(1):103. doi: 10.3390/microorganisms9010103 (PMC7824750; doi:10.3390/microorganisms9010103)
Supplement: Supplementary file 1 [file microorganisms-09-00103-s001.pdf]

Supplementary Material
for
Loss of Motility as a Non-Lethal Mechanism for Inter Colony Inhibition ("Sibling Rivalry") in *Marinobacter*

by

Ricardo Cruz-López[†], Piotr Kolesinski[†], Frederik De Boever[‡], David H. Green[‡], Mary W. Carrano[†], Carl J. Carrano[†]*

Department of Chemistry and Biochemistry

San Diego State University

San Diego, CA 92182-1030

and

Scottish Association for Marine Science

Dunstaffnage, Oban, Scotland, UK

Table S1. Sibling colony Inhibition in different strains of the *Marinobacter* genus isolated from marine environments.

sp.	Strain no.	Isolation	sibling inhibition 20°C	25°C	30°C
Marinobacter sp.	DG1136	*Gymnodinium catenatum* GC21V	-	+++	+++
Marinobacter sp.	DG1259	*Lingulodinium polyedrum* CCAP 1121/2	+	++	++
Marinobacter sp.	MH125a	*Achnanthes sp.* CCAP 1095/1	+	++	+
Marinobacter sp.	DG1239	*Scrippsiella trochoidea* CCAP 1134/1	+	++	+
M. algicola	DG893	*Gymnodinium catenatum* YC499B15	+	++	++
Marinobacter sp.	DG979	*Gymnodinium catenatum* GCTRA14	-	++	+
Marinobacter sp.	DG1194	*Amphidinium carterae* CCAP1102/7	+	+	+
Marinobacter sp.	DG1597	*Coccolithus braarudii* AC400	-	+	+
Marinobacter sp.	DG879	*Gymnodinium catenatum* GCDE08	-	+	+
Marinobacter sp.	DG1594	*Emiliania huxleyi* AC475	+	+	+
Marinobacter sp.	DG870	*Gymnodinium catenatum* GCHU11	-	+	-
Marinobacter sp.	DG1402	*Emiliania huxleyi* CCAP920/8	nd	+	nd
Marinobacter sp.	DG1205	*Gymnodinium microreticulatum* I011	nd	++	nd
Marinobacter sp.	DG1420	*Emiliania huxleyi* CCAP 920/10	nd	+	nd
Marinobacter sp.	IJ11	*Nannochloropsis* sp. (bioreactor)	nd	+	nd
M. aquaolei	VT8	Oil producing well	nd	+	nd
Marinobacter sp.	DG1623	*Skeletonema costatum* CCAP 1077/7	nd	+	nd
Marinobacter sp.	DG1305	*Scrippsiella* sp. CCMP 1073	nd	+	nd
M. lipolyticus	SM19	Saline soil	nd	+	nd
Marinobacter sp.	Pn26	*Pseudo-nitzschia fraudulenta* Pn-F	nd	-	nd
M. adhaerens	HP15	Marine organic aggregates	nd	+	nd
Marinobacter sp.	FDB33	*Nannochloropsis* sp. CCAP 849/9	nd	+	nd

-, negative; +, slight; ++, moderate; +++, high; nd, not determined.

Figure S2. Sequence analysis of the GDPD signal peptide by SignalP 5.0 server. Signal peptide likelihood was 0.718.

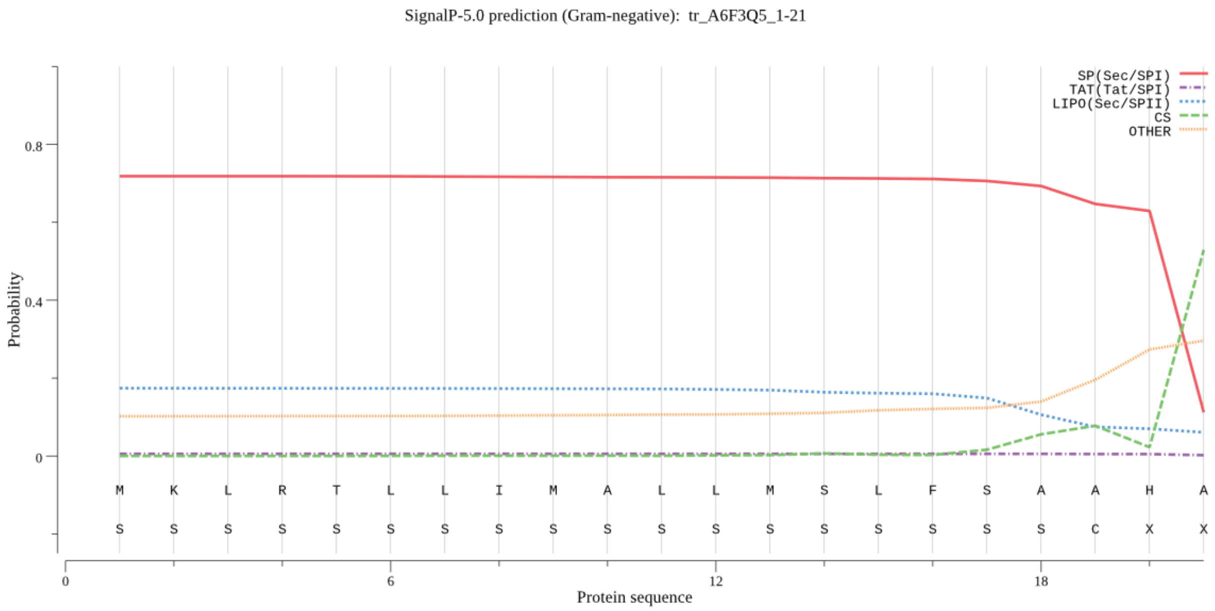

Figure S3. *M. algicola* DG893 GDPD protein-protein interaction network based on the STRING database. Ten proteins formed a network map with the GDPD. The nodes of the network (the marbles in the Figure) represent the proteins, while the edges of the network (the lines between the marbles) represent the predicted functional associations between the proteins. The color of each of the edges represents the type of evidence that exists for that interaction: a red line indicates the presence of fusion evidence, a green line indicates neighborhood evidence, a blue line indicates co-occurrence evidence, a magenta/purple line indicates experimental evidence, a yellow line indicates text-mining evidence, a light blue line indicates database evidence, and a black line indicates co-expression evidence.

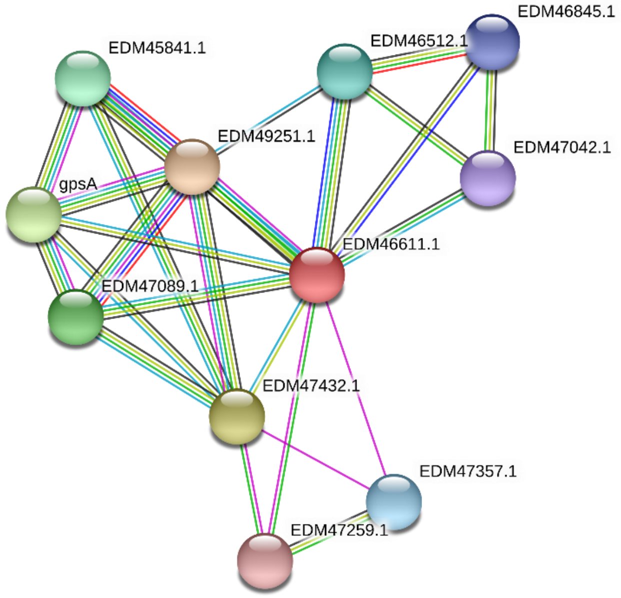

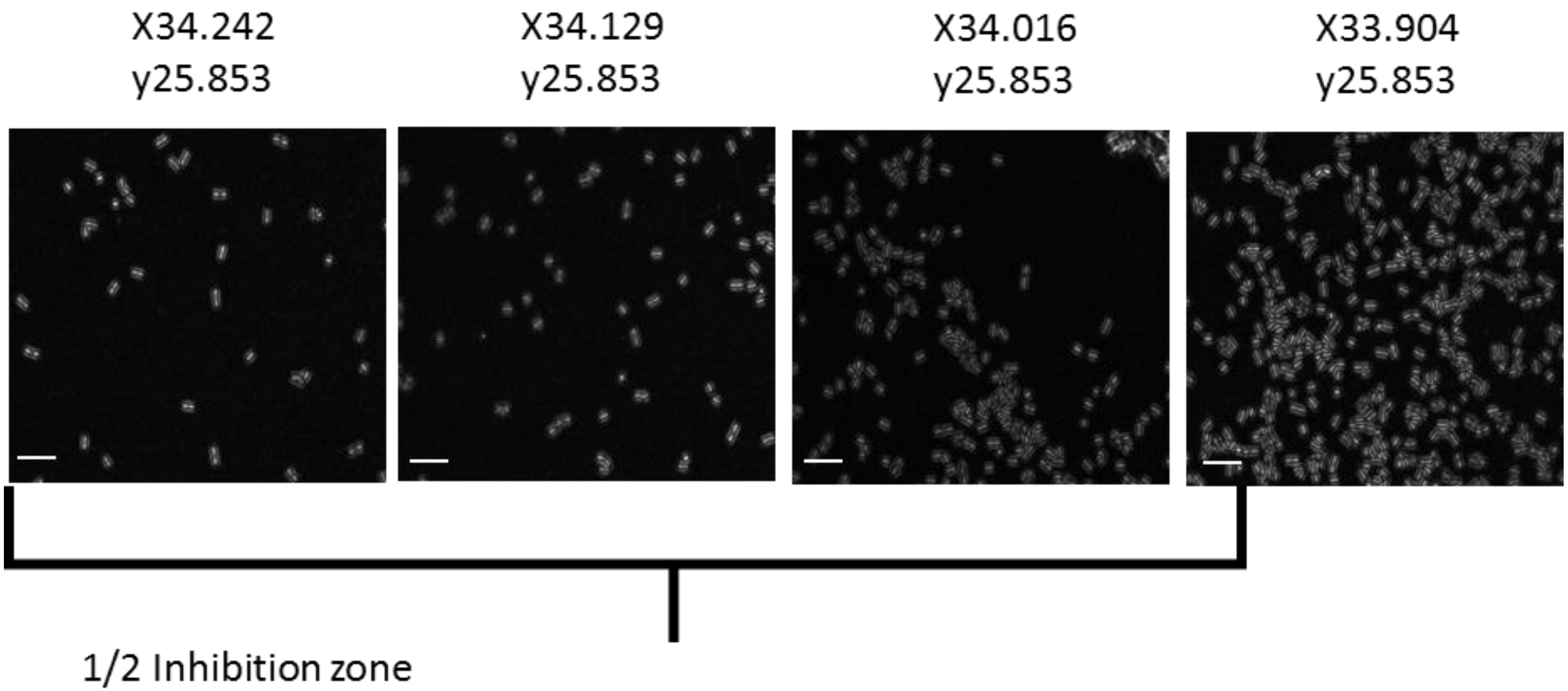

Figure S1. *M. algicola* DG893 biofilm growing on 0.3% MB agar, in a horizontal transect (x,y=mm). Scale bar, 10 μm.
